# Ten tips on how to reduce iatrogenic acute kidney injury

**DOI:** 10.1093/ckj/sfae412

**Published:** 2024-12-18

**Authors:** Hendrik Booke, Thilo von Groote, Alexander Zarbock

**Affiliations:** Department of Anaesthesiology, Intensive Care and Pain Medicine, University Hospital Münster, Münster, Germany; Department of Anaesthesiology, Intensive Care and Pain Medicine, University Hospital Münster, Münster, Germany; Department of Anaesthesiology, Intensive Care and Pain Medicine, University Hospital Münster, Münster, Germany

**Keywords:** AKI, biomarkers, CKD, dialysis, intensive care

## Abstract

Acute kidney injury (AKI) is a heterogeneous syndrome associated with worse clinical outcomes. Many treatments and procedures in the hospitalized patient can cause AKI. Hence, the incidence of iatrogenic AKI is expected to be high. In this review we provide 10 practical tips on how to manage and avoid iatrogenic AKI. We cover identification of vulnerable patients by epidemiological data and recommend the usage of renal stress biomarkers for enhanced screening of high-risk patients. Further, we discuss the limitations of current diagnostic criteria of AKI. As a key takeaway, we suggest the implementation of novel damage biomarkers in clinical routine to identify subclinical AKI, which may guide novel clinical management pathways. To further reduce the incidence of procedure-associated AKI, we advocate certain preventive measures. Foremost, this includes improvement of hemodynamics and avoidance of nephrotoxic drugs whenever possible. In cases of severe AKI, we provide tips for the implementation and management of renal replacement therapy and highlight the advantages of regional citrate anticoagulation. The furosemide stress test might be of help in recognizing patients who will require renal replacement therapy. Finally, we discuss the progression of AKI to acute and chronic kidney disease and the management of this growing issue. Both can develop after episodes of AKI and have major implications for patient co-morbidity and long-term renal and non-renal outcomes. Hence, we recommend long-term monitoring of kidney parameters after AKI.

## INTRODUCTION

**Figure 1:**
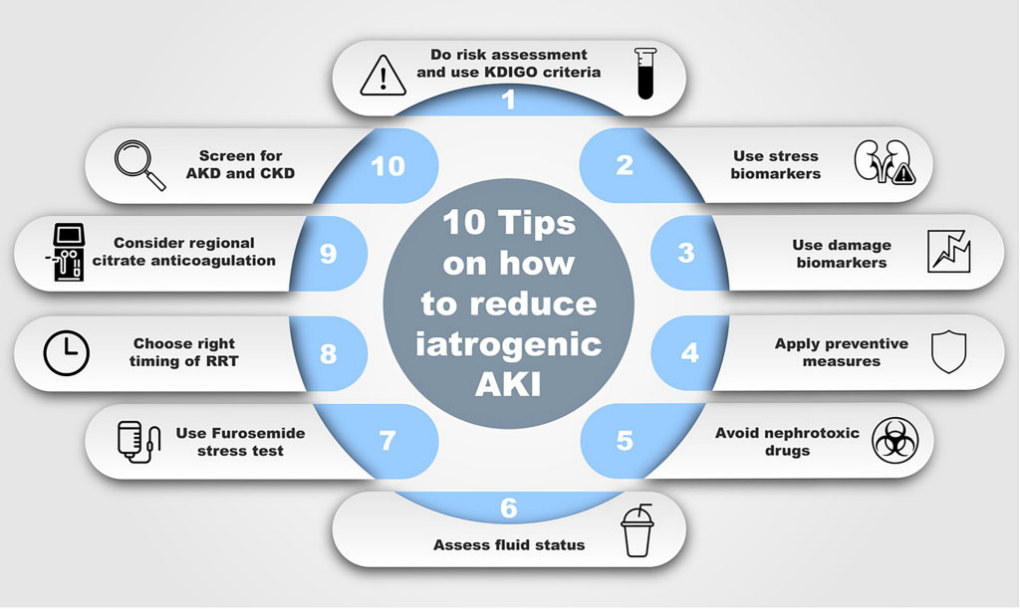
Overview of 10 suggested tips on how to reduce iatrogenic acute kidney injury (AKI). AKD, acute kidney disease; CKD, chronic kidney disease; KDIGO, Kidney Disease: Improving Global Outcomes; RRT, renal replacement therapy.

Acute kidney injury (AKI) is a heterogeneous clinical syndrome, characterized by an acute decline in kidney function [1]. AKI is diagnosed using surrogate parameters of glomerular filtration rate (GFR), namely serum creatinine (SCr) and urine output (UO) [2]. AKI can lead to complications, such as fluid overload or acid–base disorders. If AKI occurs, this is associated with worse clinical outcomes, such as increased rates of mortality, longer length of hospital stay, and development of chronic kidney disease [3, 4]. A recent international multicenter study demonstrated that 20% of all patients undergoing major surgery develop postoperative AKI [5]. With 300 million surgeries performed each year, this alone leads to a substantial number of patients with iatrogenic AKI [6]. Additionally, AKI is often underreported and the actual incidence of AKI is most likely higher than expected [3]. Still, no causal or direct treatment for AKI exists and clinicians must therefore focus on diagnosing kidney injury, prevention of AKI, and promoting early recovery. Hence, we put together 10 practical tips on how to diagnose, prevent, and manage iatrogenic AKI (Fig. 1).

## TIP 1: KNOW WHICH PATIENTS ARE AT RISK FOR AKI AND USE THE KDIGO CRITERIA FOR DIAGNOSIS

To diagnose and stage AKI, the current Kidney Disease: Improving Global Outcome (KDIGO) criteria should be applied, using SCr and UO as markers of kidney function [2]. Patients at high risk for AKI should receive frequent screening of these parameters. However, awareness of limitations of these markers is mandatory; SCr depends on muscle mass and a baseline value, necessary for AKI diagnosis, is not always available [7–9]. In addition, volume resuscitation can result in false low SCr values [10, 11]. Lastly, SCr latency is high and causes delayed diagnosis of actual injury [1]. UO on the other hand is a highly sensitive marker for detecting AKI; however, specificity is poor [12, 13]. Nevertheless, it is of high value as reduced UO alone is also associated with increased mortality [14].

Knowledge about which patients inherit a general risk for AKI can help in allocating clinical resources for thorough screening. In general, hospitalized patients can have endogenous (patient related) and exogenous (disease or procedure related) risk factors for AKI development. Risk stratification based on the combination of these factors can help in allocating screening resources. As a result, we recommend thorough screening for AKI in hospitalized patients with diseases or procedures that are closely associated with the development of AKI. Septic patients, for instance, are at very high risk for AKI, with reported rates of up to 67% [15, 16]. Patients in septic shock are even more likely to develop AKI [17]. Another clinical entity at high risk for AKI is cardiogenic shock, which holds a risk for AKI of >30% [18]. Recently, the Epidemiology of Surgery-Associated Acute Kidney Injury (EPIS-AKI) trial reported an AKI incidence of 20% after major surgery [5]. Patients who underwent urological, cardiac, vascular, and abdominal surgery were especially likely to develop postoperative AKI. In addition to these exogenous factors, age is a main endogenous risk factor for AKI. Each decade of age doubles the incidence of AKI and, on average, individuals >80 years of age have one AKI episode per year [19]. As a result, old patients who undergo major surgery could profit from close urine output and creatinine monitoring post-surgery. In a young patient with the same procedure, however, close monitoring might not be necessary as AKI diagnosis is unlikely.

Another often discussed cohort is patients who receive radiocontrast media. Radiocontrast media have a reputation of inducing AKI [20]. However, with the introduction of low or iso-osmolar radiocontrast media, the risk for AKI is probably lower than previously assumed and recent studies raised doubts about the association of AKI and contrast media application overall [21, 22]. This raises the question whether routine monitoring of SCr after radiocontrast media exposure is really necessary, which currently remains unanswered.

## TIP 2: CONSIDER USING STRESS BIOMARKERS FOR DETECTION OF PATIENTS AT HIGH RISK FOR AKI AND CCL14 TO DETECT AKI PROGRESSION

The above-mentioned risk factors can support clinical judgment. However, as mentioned above, one major limitation is that most patients inherit at least one risk factor for AKI development, which makes resource allocation extremely difficult. In addition, some patients inherit multiple risk factors and still do not develop AKI. As a result, research in the last decade focused on the discovery of other screening tools offered a variety of novel biomarkers with promising results towards earlier detection of developing AKI [23]. A differentiation of these biomarkers into damage and stress biomarkers is necessary to understand their usability. Damage biomarkers are elevated when kidney cells are damaged and indicate a direct injury of the kidneys. Stress biomarkers are elevated when kidney cells are stressed and indicate a possible loss of kidney function. Therefore, stress biomarkers have to potential to predict the development of AKI. Hence, these biomarkers could be of interest for further risk stratification. Of all stress biomarkers, TIMP-2 and IGFB7 are the most prominent in this field and indicate cell-cycle arrest. Elevated levels are associated with the development of AKI up to 48 hours later [24, 25]. Sensitivity and specificity of the [TIMP-2]•[IGFB7] test depends on the cut-off values used. A cut-off value of 0.3 ng/mL^2^/1000 showed a sensitivity of 0.72 and a specificity of 0.58, while a 2.0 ng/mL^2^/1000 cut-off value showed a 0.38 and 0.94 sensitivity and specificity, respectively [26]. Based on these analyses, the overall diagnostic accuracy for [TIMP-2]•[IGFB7] is only moderate [26]. Even though other models for assessment of AKI without biomarkers showed similar, if not even better, accuracy than biomarkers, they also have their flaws, which makes their implementation in clinical practice unlikely [27]. All tools for AKI prediction, scores and biomarkers alike, face the same problem; the gold standard for AKI diagnosis is imperfect, which influences the apparent predictive performance of these scores and biomarkers [28]. Nonetheless, renal stress biomarkers may still be of value for clinical decision-making and improve identification of patients at high risk for AKI [29]. Furthermore, values can be measured by point-of-care devices, which makes their implementation in clinical practice easy. For example, implementation in the emergency department or after major surgery might help clinicians in their decision-making on whether to transfer patients to a regular ward, observational ward, or intensive care unit. This was shown by Kane-Gill *et al*., who investigated the use of [TIMP-2]·[IGFB7] in a real-world scenario and showed a [TIMP-2]•[IGFB7]-driven impact on clinical practice [30]. Moreover, the PrevAKI and BigpAK trials showed a significant reduction in moderate and severe AKI with a biomarker-triggered intervention [31–33], and the Acute Dialysis Quality Initiative (ADQI) proposed the inclusion of biomarkers in the definition of AKI [34]. As a result, we believe that, in the future, renal stress biomarkers will be another tool in the assessment of AKI risk in certain patients. A routine implementation after major surgeries with high risk for AKI (e.g. cardiac surgery) or in patients with sepsis is thinkable to further allocate nephroprotective resources. However, as of right now, bigger and definite trials are still missing to justify the use of biomarkers as the new standard for AKI diagnostics, especially because their impact on patient-centered outcome parameters is not proven yet. We hope the multicenter BigpAK-2-trial will provide this definite answer concerning the use of biomarkers in the setting of post-surgery AKI diagnostics [35].

Novel biomarkers might also help in predicting long-term kidney trajectories. The RUBY trial showed that the C-C motif chemokine ligand 14 (CCL14) was able to predict persistent AKI in patients with new-onset moderate or severe AKI [36]. CCL14 mediates pro-inflammatory chemotaxis and plays an important role in the recruitment of monocytes and macrophages [37, 38], which are believed to have an influence on AKI development, modulating kidney fibrosis on the AKI-to-CKD continuum [39]. Subsequent studies confirmed the predictive abilities of CCL14 for the necessity of renal replacement therapy [40, 41]. However, as of right now, evidence supporting a routine measurement of CCL14 in all patients with moderate and severe AKI does not exist.

## TIP 3: USE DAMAGE BIOMARKERS FOR DETECTION OF SUBCLINICAL AKI

The aforementioned limitations of current diagnostic criteria result in a delayed detection of kidney damage. In addition, not all injuries lead to a rise in functional kidney parameters, resulting in missed diagnoses of kidney damage. Novel damage biomarkers can help with this matter by directly detecting damage of kidney cells. This led to the new term of ‘subclinical AKI’, defined by elevated damage biomarkers without elevation of functional biomarkers (SCr, UO), offering a whole new definition of renal injury [42]. Because subclinical AKI is also associated with increased mortality, The ADQI recommends the implementation of novel biomarkers in the AKI work-up [34]. Although, they do not recommend specific markers, neutrophil gelatinase-associated lipocalin (NGAL) is the most promising biomarker in this field [43]. NGAL is a damage marker, expressed by neutrophils, epithelial tissue, and tubular cells, and is elevated when kidney cells are damaged.

## TIP 4: APPLY PREVENTIVE MEASURES IN PATIENTS AT HIGH RISK

In patients at high risk for AKI, optimal clinical management may prevent the development of iatrogenic AKI. As mentioned before, identification of patients at risk is essential for precise allocation of preventive measures. The implementation of the recommendations for prevention and management of AKI, as outlined by the KDIGO 2012 AKI guidelines, might provide an effective tool for prevention of AKI [2]. Several studies demonstrated this effect in post-surgical patients with subclinical AKI (as identified by elevated urinary biomarkers [TIMP-2]•[IGFBP7]). First, the monocentric PrevAKI study randomized 276 patients after cardiac surgery to receive either standard of care or the KDIGO recommendations [31]. AKI was significantly reduced with the intervention compared with controls [55.1% vs 71.7%, absolute risk reduction (ARR) 16.6% (95% CI 5.5–27.9%), *P* = 0.004] [31]. The subsequent PrevAKI multicenter study used the same methodology in cardiac surgery patients, but found no significant difference in AKI rates (46.3% in the intervention group vs 41.5% in controls, ARR 4.8%, 95% CI −16.4 to 6.9%, *P* = 0.423) [32]. However, rates of moderate or severe AKI were significantly lower in the intervention group (14.0% vs 23.9%; ARR 10.0%, 95% CI 0.9–19.1; *P* = 0.034) [32]. Similar findings were reported in the BigpAK study by Göcze *et al*., which implemented modified KDIGO recommendations in a cohort of abdominal surgery patients [33]. Again, patients at high risk were identified by elevated [TIMP-2]•[IGFBP7] biomarker levels. Whilst AKI rates overall did not statistically differ, rates of moderate or severe AKI were significantly lower in the intervention group [6.7% vs 19.7% in the standard care group (12/60), *P* = 0.04; OR, 3.43 (1.04, 11.32)] and incidence of persistent renal dysfunction (defined as SCr increase by > 25% from baseline value) was reduced to 40.0% vs 62.3% in the standard care group [*P* = 0.01; Q8 OR, 2.48 (1.19, 5.15)]. Interestingly, the intervention was also associated with a cost reduction and shorter length of ICU and in-hospital stay. The effectiveness of the KDIGO AKI recommendations for prevention of AKI in high-risk patients (defined as [TIMP2]·[IGFBP7] positive) of all surgical disciplines is currently being investigated in the international, multicenter BigpAK-2 randomized controlled trial [35, 44].

Key aspects of the KDIGO recommendations for prevention and management of AKI in the aforementioned studies were measures to improve hemodynamics in patients at risk for AKI. As renal perfusion pressure is defined as mean (renal) arterial pressure minus mean (renal) venous pressure, ensuring adequate mean arterial pressure (MAP) while avoiding venous congestion is important. It is noteworthy that both severity and duration of hypotensive episodes are associated with AKI [45].

However, we suggest that efforts to improve hemodynamics should not only focus on MAP and central venous blood pressure, but also include evaluation and eventual optimization of cardiac output to ensure adequate renal blood flow [46]. A retrospective analysis of the combined PrevAKI-1 and PrevAKI-2 study databases found a strong association of low cardiac output and increased rates of moderate or severe AKI [47]. Interestingly, the study showed a ‘dose–effect’ relationship for mean MAP/mean CI and AKI (the lower the MAP/CI, the higher the rate of moderate/severe AKI) [48]. Improvement of low MAP or low CI was associated with lower rates of moderate or severe AKI when compared with patients in which these two measures were not improved. Furthermore, evidence exists that personalized hemodynamic management can help to avoid AKI. A retrospective analysis of septic shock patients revealed a lower AKI incidence in those patients with post-resuscitation MAP closest to baseline MAP before sepsis [49]. The INPRESS trial showed that an individualized blood pressure target during major surgery may result in a reduction of renal dysfunction [50]. However, further evidence regarding personalized approaches is needed.

Finally, evidence suggests that the implementation of advanced hemodynamic monitoring and assessment of volume status reduces AKI occurrence and this is also part of the aforementioned KDIGO AKI care bundle [51].

## TIP 5: AVOID NEPHROTOXIC DRUGS IF POSSIBLE OR APPLY THERAPEUTIC DRUG MONITORING TO LIMIT DRUG NEPHROTOXICITY

Another common cause of AKI is drug nephrotoxicity. For example, in critically ill patients, one-quarter of AKI cases are expected to be drug-related [52]. Many drugs like antimicrobials (e.g. vancomycin), chemotherapeutics (e.g. cisplatin), and analgesics [e.g. non-steroidal anti-inflammatory drugs (NSAIDs)] are potentially nephrotoxic [53]. Nephrotoxicity of these drugs acts by different pathomechanisms. Some drugs (e.g. aminoglycosides and amphotericin B) cause direct injury to kidney cells, while others (e.g. β-lactams and NSAIDs) lead to tubulointerstitial inflammation with following kidney injury. Drugs like acyclovir, methotrexate and indinavir/atazanavir are insoluble in the tubular system and cause crystalline nephropathy [53]. Combinations of drugs can further aggravate their toxic side effects (e.g. vancomycin and piperacillin–tazobactam) [54].

As a result, the KDIGO recommends avoidance of nephrotoxic drugs in patients at high risk for AKI, if possible. If avoidance or discontinuation of such drugs is not possible, therapeutic drug monitoring-guided dosing can limit nephrotoxicity [2].

## TIP 6: ASSESS FLUID STATUS OF PATIENTS WITH AKI AND PATIENTS WHO ARE AT RISK FOR AKI

We recommend monitoring of fluid status in patients with AKI or at risk for AKI. This is mandatory for a patient-individualized approach that equally avoids extensive volume overload and hypovolemia [55]. In addition to daily fluid balance, ultrasound of the inferior vena cava diameter and variance, as well as performance of the passive leg-raising test can be used to evaluate fluid status and possible fluid responsiveness [56, 57]. Another way to assess fluid status using ultrasound is the VExUS score [58]. It is a four-dimensional score evaluating venous congestion by assessing vena cava diameter, hepatic vein Doppler, portal vein Doppler, and renal vein Doppler [58]. Higher scores are associated with the development of AKI [58].

For treatment of hypovolemia, different kinds of fluids are available and studies investigating the optimal fluid in AKI management are ongoing. In general, fluids are divided into crystalloids and colloids. Colloids are large molecules within a solution, which do not cross the endothelial membrane as much as crystalloids do [59]. This promises a better volume expansion with reduced distribution into the extravascular space. However, this assumption may be wrong in the setting of lost vascular barrier integrity, commonly observed in the perioperative setting and sepsis.

Because of that and various other reasons, crystalloids are generally preferred for volume resuscitation in patients with or at risk of AKI [60–66]. Studies investigating normal saline vs balanced solutions as resuscitation fluids showed no effects regarding mortality and only little effect regarding major adverse kidney events on day 30 [67]. However, the patients in these trials often required only small amounts of fluids, which questions the generalizability of these results [68–70]. In addition, as normal saline is hypertonic, acidic (induces hyperchloremic dilutional acidosis), and associated with renal vasoconstriction, the authors personally prefer balanced solutions as their standard fluid for resuscitation [59, 71, 72].

Furthermore, it is unclear whether a more restrictive or liberal fluid management regime is favorable. For instance, the Conservative versus Liberal Approach to Fluid Therapy in Septic Shock (CLASSIC) study group showed no difference in AKI occurrence between a restrictive and a liberal fluid regime in patients with septic shock [73]. The POINCARE-2 study had similar results regarding AKI in the ICU setting [74, 75]. However, in another randomized controlled trial with patients on continuous renal replacement therapy (CRRT), a positive fluid balance was associated with increased mortality [76]. But, in the setting of major surgery, a positive fluid balance showed decreased rates of AKI [77]. As of now, there is no ‘one size fits all’ strategy for fluid management in AKI and patient individual factors need consideration in the clinician's regime.

## TIP 7: APPLY THE FUROSEMIDE STRESS TEST TO IDENTIFY PATIENTS THAT MAY BENEFIT FROM RENAL REPLACEMENT THERAPY

Early identification of patients who will require renal replacement therapy (RRT) is crucial for timely intervention and improved prognosis. The furosemide stress test (FST), involving the administration of furosemide and monitoring the subsequent urine output, has gained attention as a predictive tool. Based on the initial publication by Chawla *et al*., the FST is usually performed by infusing 1 mg furosemide/kg ideal body weight (IBW) in furosemide-naive patients (this may be escalated to up to 1.5 mg/kg IBW in patients that received loop diuretics before) [41]. Subsequently, the response of urinary output to furosemide within 2 hours after the FST can be indicative of the remaining functional nephron mass and tubular integrity. Patients with significant tubular damage exhibit a blunted diuretic response and a urine output of <200 mL in these 2 hours is considered a poor response, indicating a high likelihood of requiring RRT. Chawla *et al*. found that a negative FST was strongly associated with progression to stage 3 AKI and the need for RRT.

The application of the FST yields a promising new tool in the management of patients with AKI, given its simplicity, low cost, and rapid result. Also, diuretic-related complications, such as hypovolemia, hypokalemia, or transient hearing loss, are unlikely with the dosages used. However, further research is required to improve understanding in optimal dosing.

Moving forward, combining the FST with biomarkers of AKI progression may improve the predictive performance of the FST even further. In a monocenter cohort study, the combination of te FST and CCL14 predicted the development of an absolute RRT indication with an area under the receiver operating characteristic of 0.87 (95% CI 0.82–0.92), which was better than the FST or biomarker alone [41].

## TIP 8: CHOOSE THE RIGHT STARTING POINT FOR RENAL REPLACEMENT THERAPY

Conventional indications for RRT in AKI include acidosis, severe electrolyte dysbalance, fluid overload, and uremia [78]. In most cases, a central venous catheter capable of adequate blood flow is required for CRRT. The KDIGO recommends the right internal jugular vein as the standard insertion site, followed by the femoral veins and the left internal jugular vein. If all of these are inadequate (e.g. due to thrombosis), the subclavian vein can be used for catheter placement. In this case, the dominant side is preferred. The non-dominant site should be spared if possible, in case a shunt is required for permanent dialysis dependency [2].

Different modalities of RRT are available. In general, treatments are divided into continuous (CRRT) and intermittent (intermittent hemodialysis, IHD). CRRT stretches the dialysis volume over the entire day and has therefore only little impact on hemodynamics. IHD, however, removes fluids over a shorter period and can lead to decreases in blood pressure. On the contrary, IHD allows better mobilization and patient comfort. Hybrid forms, e.g. sustained low-efficiency daily dialysis (SLEDD), are possible. In patients who cannot be treated with hemodialysis at all (e.g. in low-resource settings), intermittent peritoneal dialysis is another option for treatment of dialysis requiring AKI [79].

For CRRT, the KDIGO recommends a delivered effluent dose of 20–25 mL/kgKG/h. Because of downtimes (e.g. procedures) a higher prescription of around 30 mL/kgKG/h is needed [2].

Life-threatening conditions that can be resolved by RRT warrant immediate initiation of dialysis [2]. The optimal timing of RRT in non-life-threatening conditions was the subject of many randomized controlled trials. The Effect of Early vs Delayed Initiation of Renal Replacement Therapy on Mortality in Critically Ill Patients With Acute Kidney Injury (ELAIN) trial showed a survival benefit of early (moderate AKI + elevated NGAL) RRT initiation [80]. Contrary to this result, the STARRT-AKI, AKIK, and IDEAL-ICU studies showed no benefit of an early approach [48–50]. Moreover, the AKIKI study showed a high rate of avoided RRT in the delayed group. As a result, the AKIKI-2 study compared a delayed with a more-delayed RRT approach in patients with AKI, but showed more adverse events in the more-delayed group [81]. However, comparison of these studies is difficult. For instance, the ELAIN trial included mostly surgical patients, whereas the IDEAL-ICU study included septic patients. Furthermore, the definition of early and delayed differed substantially. Subsequently, a generalized recommendation on the right timing of RRT might not be possible and clinical judgment, although difficult at times, should be an important part of decision-making. Further research and new methods, like the above-mentioned FST, could aid clinicians in their decision. Right now, in the majority of patients a watchful waiting approach for initiation of RRT seems best in order to avoid unnecessary RRT exposure.

## TIP 9: CONSIDER USING REGIONAL CITRATE ANTICOAGULATION FOR CRRT WHENEVER POSSIBLE

We advocate regional citrate anticoagulation over systemic anticoagulation for continuous renal replacement therapy. Anticoagulation is recommended for (C)RRT to ensure filter patency [82]. For a long time, systemic anticoagulation using heparin or no anticoagulation in combination with high blood flow were the standard anticoagulation regimens for RRT [83]. With the introduction of regional citrate anticoagulation (RCA) this management was challenged concerning patient outcome and filter lifespan. Citrate is added to the extracorporeal circuit at the beginning of the circuit and inhibits blood coagulation via binding of calcium ions (factor IV of the coagulation cascade). At the end of the circuit, calcium is added with a syringe pump to ensure normal intracorporeal calcium levels. As a result, blood coagulation of patients with regional citrate RCA RRT is not compromised and the risk of bleeding or other heparin-induced complications, such as heparin-induced thrombocytopenia, is reduced [84]. At the same time, filter lifespan is improved with the regional anticoagulation strategy [84]. However, RCA requires close monitoring of intracorporeal and extracorporeal calcium levels. Further, the added citrate solutions can cause acid–base disturbances. Sodium content in these solutions is usually high and causes an increase in strong ion difference, which again leads to alkalosis. Excessive citrate administration is also described as citrate overload and is a benign complication of RCA [85]. In this case, all citrate–calcium complexes are metabolized and the amount of ionized calcium is normal. Another possible complication is accumulation of citrate. This occurs when citrate metabolism is saturated. This leads to unmetabolized citrate, hypocalcemia, and high anion gap metabolic acidosis. Altogether, this causes decreased myocardial contractility with vasoplegia and possible death. As citrate is cleared by the hemofilter/dialysate and remaining citrate is metabolized in the liver, muscle, and kidneys, citrate accumulation can occur with insufficient dialysate flow, early membrane clogging, and reduced metabolism. Most common reasons for reduced metabolism are impaired liver function and circulatory shock. But, with close monitoring, even in severe liver failure safe use of RCA is possible [86]. We recommend daily assessment of the total/ionized calcium ratio for detection of citrate accumulation. A ratio of >2.5 is an indicator of present accumulation and a trend towards this cut-off or increased demand for calcium substitution can also be an early warning [85]. As metabolism of citrate is oxygen-dependent, sufficient cellular respiration is important to avoid citrate accumulation, and elevated lactate levels should also be a trigger for cessation of RCA [86]. Another complication of RCA might be higher rates of infection, as data suggest [84]. However, reasons for this effect are unclear and currently under further investigation [87]. Nevertheless, the KDIGO recommends use of regional anticoagulation whenever possible as the benefits outweigh the rarely occurring and avoidable complications [2]. For patients with systemic anticoagulation because of extracorporeal membrane oxygenation (ECMO) the authors also use regional anticoagulation whenever possible, as, compared with ECMO, RRT blood flow is lower and RRT tubes are typically not heparin-coated. In a retrospective analysis, this strategy resulted in longer filter lifespan in a cohort of veno-venous ECMO patients [88].

## TIP 10: SCREEN PATIENTS FOR ACUTE KIDNEY DISEASE AND CHRONIC KIDNEY DISEASE AFTER AKI

Besides short-term outcomes, AKI can also lead to long-lasting disturbances of renal function [3, 4, 89]. Episodes of reduced kidney function which persist for >7 days are classified as acute kidney disease (AKD); and after >90 days the term chronic kidney disease (CKD) is used [90]. Today, CKD affects >400 million patients globally and is a major risk factor for cardiovascular and cerebrovascular diseases, causing an immense burden for patient quality of life and healthcare systems [91]. It is known that AKI and CKD are linked with one another and that AKI is a major risk factor for the development of CKD [89]. As mentioned before, one promising biomarker already in use for projecting long-term outcomes of patients with AKI is CCL14. Elevated levels of CCL14 in patients with AKI grade 2 or 3 hold predictive value for persistent loss of renal function and renal non-recovery [36, 92, 93]. Whether CCL14 can be used to allocate resources and hinder the transition to persistent AKI and the need for dialysis is currently under investigation (NCT05275218).

As a consequence of the possible AKI-to-CKD transition, the KDIGO practice guidelines recommend re-evaluation of patients 3 months after AKI [2]. However, no specific recommendations were made regarding the extent of follow-up care and needed specialties. The effects of AKI involve many medical specialties (intensivists, nephrologists, cardiologists etc.) and hence the patients may benefit from multidisciplinary follow-up-care. However, there is currently no evidence regarding structured post-AKI work-up. We therefore recommend that patients with episodes of AKI should primarily receive post-AKI work-up, of any kind, to detect a possible AKI-to-CKD transition. In a second step, referral to a specialist might be beneficial to prevent further transition to AKD/CKD with all its consequences.

However, some patients also develop AKD without episodes of AKI [94]. Currently, the pathomechanisms behind this slower loss of kidney function are not understood. One possible explanation may be that the functional loss caused by minor damage of the kidneys does not necessarily fulfil KDIGO criteria. Similar to subclinical AKI having implications for short-term patient outcome, multiple instances of subclinical AKI may slowly lead to AKD or CKD [42, 95]. Long-term effects of renal damage without immediate loss of function will be the subject of further investigations with a focus on early detection.

## CONCLUSIONS

AKI impacts patient outcomes, but at the same time current diagnostic criteria have significant limitations. Novel biomarkers can help in detecting kidney damage and to identify patients at high risk for AKI. Preventive measures can lower the incidence of iatrogenic AKI. In patients with AKI, establishing an optimal environment (hemodynamics, fluid status, and medication) is crucial to enable kidney recovery. If RRT is needed, regional citrate anticoagulation is favored. Neither early nor late RRT is recommended and decision-making should be made based on the indication for RRT. If AKI occurs, long-term follow-up should be initiated to avoid development of CKD.

## Data Availability

No new data were generated or analyzed in support of this research.
